# Mitochondrial Haplogroups, Control Region Polymorphisms and Malignant Melanoma: A Study in Middle European Caucasians

**DOI:** 10.1371/journal.pone.0027192

**Published:** 2011-12-09

**Authors:** Sabine Ebner, Roland Lang, Edith E. Mueller, Waltraud Eder, Michaela Oeller, Alexandra Moser, Josef Koller, Bernhard Paulweber, Johannes A. Mayr, Wolfgang Sperl, Barbara Kofler

**Affiliations:** 1 Research Program for Receptor Biochemistry and Tumor Metabolism, Department of Pediatrics, Paracelsus Medical University, Salzburg, Austria; 2 Department of Dermatology, Paracelsus Medical University, Salzburg, Austria; 3 Department of Internal Medicine, Paracelsus Medical University, Salzburg, Austria; Maastricht University Medical Center, Netherlands

## Abstract

**Background:**

Because mitochondria play an essential role in energy metabolism, generation of reactive oxygen species (ROS), and apoptosis, sequence variation in the mitochondrial genome has been postulated to be a contributing factor to the etiology of multifactorial age-related diseases, including cancer. The aim of the present study was to compare the frequencies of mitochondrial DNA (mtDNA) haplogroups as well as control region (CR) polymorphisms of patients with malignant melanoma (n = 351) versus those of healthy controls (n = 1598) in Middle Europe.

**Methodology and Principal Findings:**

Using primer extension analysis and DNA sequencing, we identified all nine major European mitochondrial haplogroups and known CR polymorphisms. The frequencies of the major mitochondrial haplogroups did not differ significantly between patients and control subjects, whereas the frequencies of the one another linked CR polymorphisms A16183C, T16189C, C16192T, C16270T and T195C were significantly higher in patients with melanoma compared to the controls. Regarding clinical characteristics of the patient cohort, none of the nine major European haplogroups was associated with either Breslow thickness or distant metastasis. The CR polymorphisms A302CC-insertion and T310C-insertion were significantly associated with mean Breslow thickness, whereas the CR polymorphism T16519C was associated with metastasis.

**Conclusions and Significance:**

Our results suggest that mtDNA variations could be involved in melanoma etiology and pathogenesis, although the functional consequence of CR polymorphisms remains to be elucidated.

## Introduction

Malignant melanoma is one of the most aggressive skin cancers arising from the pigment-producing cells called melanocytes. Due to the fact that its worldwide incidence in Caucasian populations has been rising steadily for several decades [1], malignant melanoma has become an important public health issue and its early detection remains vital to lowering mortality [2]. As in many other malignancies, both genetic predisposition and environmental risk factors such as UV radiation are involved in melanoma development [1].

Mitochondria play an essential role in energy metabolism, initiation of apoptosis and generation of reactive oxygen species (ROS). Therefore, it has long been postulated that variation of mitochondrial functions may contribute to the development and progression of cancer [3].

Mitochondria possess their own DNA (mtDNA), a circular double-stranded molecule consisting of 16,569 base pairs coding for 37 genes: 13 protein subunits of the electron transport chain, 22 tRNAs and 2 rRNAs. In addition, mtDNA contains a non-coding control region (CR), also called the D-loop, with regulatory sequences controlling mtDNA replication and transcription [4]. The mutation rate of mtDNA is approximately 10-fold higher than that of nuclear DNA. This is due to the absence of protecting histones and the lack of an efficient DNA repair system in mitochondria. Furthermore, the respiratory chain is a potent source of free radicals, which additionally can lead to DNA damage [3], [5]. It has been hypothesized that mtDNA mutations or inherited polymorphisms may alter the encoded protein subunits of the respiratory chain complexes. This in turn could result in altered ROS production, precipitating a cascade of events, including elevated levels of mtDNA mutation, impairment of respiratory chain activity, and further ROS generation, thus setting in motion a vicious circle of oxidative stress, which may be involved in the formation and progression of tumors [6], [7], [8].

Somatic mtDNA mutations have been found in several malignancies, including breast, ovarian, endometrial, prostate, colorectal, gastric, thyroid, renal, hepatocellular, esophageal, pancreatic and brain tumors [3], [5], [9]–[12]. In particular, the D-loop region (nucleotides 16024–516) has been shown to be a mutational hot spot in human malignancies [13], [14]. There is strong evidence that genetic instability in the D-loop region may be involved in carcinogenesis of human cancers, possibly by affecting copy number and gene expression of the mitochondrial genome [15].

Mithani et al. reported finding somatic mitochondrial mutations in melanoma specimens and melanoma cell lines, with an incidence of 75% [16]; the incidence of mutations within the D-loop region was considerably higher than for the remainder of the mitochondrial genome. Another study detected somatic mitochondrial mutations in 45% of melanoma cell lines and 42% of melanoma specimens [17]. Somatic alterations within the polycytidine (poly-C) tract (nucleotides 303–315) of the D-loop region were detected in 30% of melanoma cell lines and in 17% of melanomas. Deichmann et al. found several alterations of the mitochondrial D-loop in primary melanoma tumors; the overall frequency was 12% [18]. This is in line with another melanoma study, which detected an overall frequency of D-loop alterations of 13% [19].

Investigation of somatic mitochondrial mutations in cancer is one approach to assess the contribution of mtDNA variation to melanoma development and progression. The other approach is to examine disease-associated mtDNA haplotypes and single-nucleotide polymorphisms (SNPs). There is evidence that “neutral” germ-line mtDNA polymorphisms may be risk factors for age-related multifactorial diseases, including cancer, and that they may influence disease outcome [20], [21]. Association with mitochondrial haplogroups has been discussed for prostate and renal cancer [22], breast [21], [23], thyroid [24], esophageal [25], endometrial [26] and colorectal cancer [27].

The aim of the present study was to compare the frequencies of mtDNA haplogroups and CR polymorphisms of patients with malignant melanoma to those of healthy controls in Middle Europe.

## Results

The nine major European mtDNA haplogroups and CR polymorphisms were analyzed in whole blood samples of 351 patients with malignant melanoma and compared to 1598 control subjects [28]. Clinical characteristics of the patients and controls are shown in Table 1.

**Table 1:** Characteristics of the study populations.

	Patients with melanoma	Controls
	n = 351	n = 1598
Mean (SD^1^) age (years)	59.2 (16.2)	51.7 (6.1)
Male (%)	49.0	63.9
Breslow thickness		
≤1.00 mm	n = 154 (43.9%)	n.a.
1.01–2.00 mm	n = 89 (25.4%)	n.a.
2.01–4.00 mm	n = 53 (15.1%)	n.a.
>4.00 mm	n = 32 (9.1%)	n.a.
missing	n = 23 (6.5%)	n.a.
Metastasis		
yes	n = 66 (18.8%)	n.a.
no	n = 276 (78.6%)	n.a.
missing	n = 9 (2.6%)	n.a.

1 SD: standard deviation.

n.a.: not applicable.

### MtDNA haplogroup distribution in patients with malignant melanoma

The frequencies of the major European mitochondrial haplogroups did not differ significantly between patients with melanoma and control subjects (Table 2). Only the frequency of haplogroup K was significantly lower in patients with malignant melanoma (p = 0.043), but the difference did not remain statistically significant after adjustment for sex and age (p = 0.129).

**Table 2:** Frequencies (%) of Caucasian mitochondrial haplogroups in cases and controls.

Haplogroup	Patients with melanoma	Controls	p-Value^1^
	(n = 351)	(n = 1598)	
H	41.9	44.0	0.470
U	16.8	15.2	0.452
J	10.5	11.1	0.746
T	9.1	8.4	0.657
K	2.9	5.5	0.043
W	2.3	2.1	0.800
V	1.7	1.7	0.979
I	1.4	0.9	0.385
X	1.4	1.4	0.983
Others^2^	12.0	9.7	0.202

1 p-Value: Pearson chi-square or Fisher's exact test.

2 Haplogroups that could not be assigned to one of the nine major European haplogroups.

### CR polymorphisms in patients with malignant melanoma

The second part of our study focused on CR polymorphisms. MtDNA was analyzed between nucleotide positions (np) 16024 and 526 in all 351 melanoma patients, and 211 homoplasmic polymorphisms were found (Table S1). Among these polymorphisms, we detected 196 single base-pair exchanges, seven single base-pair deletions and two single base-pair insertions, compared to the revised Cambridge Reference Sequence. At position 302 we found CC-insertions, and at position 310 TC-insertions in addition to C-insertions. A CA-deletion at positions 514 and 515 occurred 24 times. CA-insertions, CACA-insertions and CACACA-insertions at this site were observed 23 times. Of the 211 polymorphisms detected, 13 are not listed in MITOMAP or the Human Mitochondrial Genome Database (www.mitomap.org; www.genpat.uu.se/mtDB/). Twenty-six of the 211 CR polymorphisms were detected at a frequency ≥5% in either the melanoma or the control group [29] (Table 3). These were subjected to further statistical analysis. Five of them, A16183C, T16189C, C16192T, C16270T and T195C, were highly linked to one another and found to have a significantly higher frequency in patients with melanoma compared to the control cohort (p<0.05) (Table 3). These differences remained significant after adjustment for sex and age.

**Table 3:** Frequencies (%) of CR polymorphisms higher than 5% in either patients with melanoma or controls and odds ratios (OR) for the association between genetic variation and disease state.

mtDNA CR polymorphisms	Frequency in patients with melanoma (%)	n^1^	Frequency in controls (%)	n^1^	p-value^2^	OR^3^ (95%CI^4^)	p-value^5^	OR (95%CI)^5^
G16145A	5.1	18	4.3	68	0.471			
A16183C	5.7	20	2.2	35	<0.0005	2.70 (1.5–4.7)	0.0017	2.69 (1.4–5.0)
T16189C	16.8	59	11.9	190	0.012	1.50 (1.1–2.1)	0.045	1.42 (1.0–2.0)
C16192T	9.7	34	6.0	96	0.012	1.68 (1.1–2.5)	0.019	1.70 (1.1–2.6)
C16256T	7.7	27	6.1	98	0.280			
C16270T	12.5	44	7.8	125	0.004	1.69 (1.2–2.4)	0.030	1.55 (1.0–2.3)
C16294T	10.0	35	9.1	146	0.625			
T16304C	6.8	24	7.8	125	0.530			
T16311C	10.3	36	13.8	221	0.073			
T16362C	5.1	18	6.8	109	0.245			
A16399G	6.0	21	4.2	67	0.144			
T16519C	63.5	223	66.0	1055	0.375			
A73G	54.7	192	54.3	867	0.879			
T146C	10.3	36	9.2	147	0.539			
C150T	11.1	39	11.3	180	0.935			
T152C	23.6	83	22.7	363	0.707			
G185A	6.3	22	5.7	91	0.677			
T195C	21.9	77	17.1	273	0.032	1.36 (1.0–1.8)	0.043	1.37 (1.0–1.9)
T204C	5.1	18	4.3	68	0.471			
G228A	6.8	24	6.1	97	0.589			
C295T	10.5	37	10.3	165	0.904			
A302C-Ins	39.6	139	38.2	610	0.618			
A302CC-Ins	12.0	42	11.7	187	0.890			
T310C-Ins	98.3	345	96.9	1548	0.149			
C462T	8.0	28	8.3	133	0.831			
T489C	12.3	43	11.5	184	0.697			

1 n: number of individuals with the respective polymorphism.

2 p-value: derived from Pearson chi-square or Fisher's exact test.

3 OR: Odds Ratio.

4 CI: Confidence Interval.

5 adjusted for age and sex.

### Analysis of clinical parameters

To determine whether haplogroups or CR polymorphisms within the melanoma cohort were associated with tumor invasiveness or disease progression, we also analyzed the frequencies of mtDNA haplogroups and CR polymorphisms in relation to Breslow thickness of the tumor and metastasis.

The vertical depth of the melanoma (measured downward from the top of the stratum granulosum of the epidermis) has been shown to be the factor that best correlates with prognosis [2]. Several studies showed that Breslow thickness is associated with male gender [30], [31]. This finding was confirmed by our study. Males (n = 158) had a mean Breslow thickness of the tumor of 1.98 mm (SD 2.137) whereas females (n = 170) showed a mean of 1.50 mm (SD 1.521) (p = 0.032).

**Figure 1:**
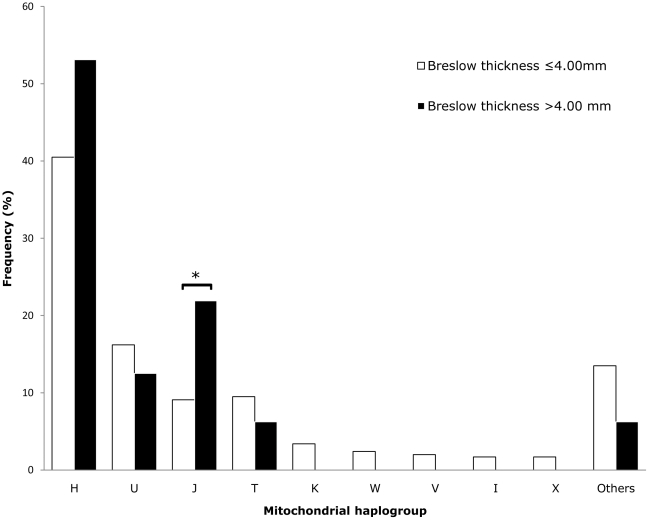
Frequencies (%) of mitochondrial haplogroups within different categories of Breslow thickness.

Logarithmized data of Breslow thickness were analyzed using a two-sample unpaired t-test. None of the nine major European haplogroups was found to be associated with Breslow thickness of melanoma (data not shown). When subdividing melanoma cases into categories for comparison of less invasive and thick melanoma (category 1: Breslow thickness ≤4.00 mm, n = 296; category 2 Breslow thickness >4.00 mm, n = 32), haplogroup J was overrepresented among thick melanomas (21.9% versus 9.1%; p = 0.034) (Figure 1). This trend of higher incidence of haplogroup J within Breslow category 2 was very similar in both sexes (data not shown). In addition, the incidence of haplogroup H rose from 40.5% (category 1) up to 53.1% (category 2) but did not reach statistical significance.

When analyzing CR polymorphisms and logarithmized data of Breslow thickness using a two-sample unpaired t-test, two polymorphisms reached statistical significance. These two variants, namely a CC-insertion at position 302 and a C-insertion at position 310, are both located within a homopolymeric C-stretch between np 303 and 315 interrupted by a thymine at position 310. This poly-C stretch is situated in the second hypervariable region (HVR II) and was reported to be a mutational hotspot [32]. Subjects harboring the A302CC-insertion (n = 41) showed a geometric mean Breslow thickness of 1.55 mm, whereas patients without this alteration (n = 287) had a geometric mean Breslow thickness of 1.07 mm (p = 0.008). In contrast, the T310C-insertion was associated with lower geometric mean Breslow thickess (1.10 mm versus 3.06 mm; p = 0.015). These two polymorphisms also reached significance when compared within the two Breslow category groups: 28.1% of patients in category 2 had the A302CC-insertion compared to 10.8% in category 1 (p = 0.002). The frequency of the T310C-insertion was 99.0% in category 1 versus 90.6% in category 2 (p = 0.020). In addition, the frequency of an A to G transition at np 73 decreased from 56.1% in Breslow category 1 to 37.5% in category 2 (p = 0.028) (Table 4).

**Table 4:** Frequencies (%) of the CR polymorphisms A73G, A302CC-Ins and T310C-Ins in Breslow categories 1 and 2.

CR polymorphism	Frequency (%) in Breslow category 1 (≤4.00 mm)	Frequency (%) in Breslow category 2 (>4.00 mm)	p-value*
A73G	56.1	37.5	0.028
A302CC-Ins	10.8	28.1	0.002
T310C-Ins	99.0	90.6	0.020

*adjusted for age and sex.

Analyses of mitochondrial haplogroups and metastasis did not reveal an association between specific haplogroups and tumor invasiveness. Concerning CR polymorphisms, the T16519C substitution was found in 48.5% of patients with metastases, compared to 34.4% without metastases (p = 0.026).

## Discussion

Because mitochondria play a central role in energy and ROS production, mtDNA is an obvious candidate for genetic susceptibility studies in cancer. In the present study, for the first time, frequencies of mtDNA haplogroups and CR polymorphisms were determined in patients with malignant melanoma. No significant difference between haplogroup frequencies of patients with melanoma and control subjects could be found. Analysis of clinical characteristics revealed that there is also no association between mtDNA haplogroups and tumor invasiveness or metastatic progression. Only when categorizing Breslow thickness did we find an association: haplogroup J was overrepresented in thick melanoma, indicating that this haplogroup might be associated with higher tumor thickness and therefore less favorable prognosis [2], [33]. However, this finding must be considered as tentative given the small sample size of patients within Breslow category 2. Therefore, further investigations should examine a higher number of melanoma cases with high values of Breslow thickness. In addition, it has to be considered that we performed a high number of statistical comparisons, which increases the possibility of obtaining significant p-values by chance.

To our knowledge, this is the first association study on melanoma and mitochondrial CR polymorphisms. To date, mutations within the D-loop are of unclear relevance, but they may influence the level of transcription and/or replication of the mitochondrial genome [5].

In the present study, we evaluated CR germline polymorphisms of the mtDNA of melanoma patients as possible risk factors for melanoma formation and progression, taking into account clinicopathological data.

The T16189C variant in the CR region has been associated with several other multifactorial disorders [29], [34], [35], including endometrial cancer [36]. The T to C substitution at position 16189 frequently generates an uninterrupted poly-C tract (np 16180–16195) in the D-loop. Furthermore, this variation often leads to heteroplasmic length variation of the poly-C tract (>10 cytosines) in different mtDNA molecules of a single person [29], [37]. When the T16189C polymorphism is accompanied by a second nucleotide change, which is often the case at np 16192, the poly-C is interrupted again. Liou et al. showed that different poly-C variants showed differences in mean mtDNA copy numbers [38]. Subjects with an uninterrupted poly-C had the lowest mtDNA copy number, whereas subjects harboring an interrupted poly-C showed the highest mtDNA copy number. These findings support an earlier assumption that the T16189C variant may affect mtDNA replication [39] because np 16189 is very close to the termination-associated sequence of the D-loop [40]. Within our melanoma cohort, the variant with the interrupted poly-C showed significantly elevated frequencies in melanoma cases compared to controls (p = 0.001). We assume that this variant may alter mean mtDNA copy number also in melanoma cells. Concerning the T195C and C16270T polymorphisms, it is unclear whether these polymorphisms may be involved in tumor formation or disease progression as there are, to our knowledge, no data available showing their effect on mtDNA replication and transcription.

We further analyzed Breslow thickness of the tumor and metastasis to determine whether a certain haplogroup or CR polymorphism within the melanoma cohort correlates with tumor invasiveness or disease progression. The A302CC-insertion, which we found to be associated with a higher mean Breslow thickness, and the T310C-insertion, which was related to lower mean Breslow values, are both located within a poly-C stretch of HVR II. This C-stretch is of interest because it is involved in the formation of the persistent RNA-DNA hybrid that leads to the initiation of mtDNA heavy-strand replication [41]. Therefore, alterations of this region, in analogy to the T16189C variant, might have an impact on transcription and replication of the mitochondrial genome. The T16519C substitution was found at a higher frequency in patients with malignant melanoma and metastases, indicating a potential link to disease progression. Previously, this polymorphism was found to be associated with increased breast cancer risk [23] and with worse prognosis in pancreatic cancer [42].

In conclusion we report for the first time an association of mtDNA variations and malignant melanoma and its clinical parameters, Breslow thickness and metastasis. Therefore, genetically determined variation in mitochondrial function has to be considered, among other factors, as a potential contributor to malignant melanoma development.

## Methods

### Ethics Statement

The study was conducted according to the Austrian Gene Technology Act and complied with the Declaration of Helsinki. All subjects gave written informed consent before entering the study. The SAPHIR program was approved by the Local Province of Salzburg Ethics Committee (“Ethikkommission für das Bundesland Salzburg; Amt der Salzburger Landesregierung, Abteilung 9 Gesundheit und Sport”).

### Patients and control subjects

Whole blood samples from 351 unrelated middle European Caucasians with malignant melanoma were recruited at the Department of Dermatology of the Paracelsus Medical University, Salzburg, Austria from April 2007 until March 2010. Patients with either melanoma in situ or melanoma in regression were excluded from the study.

The control population consisted of 1598 unrelated individuals, as previously described in detail [28], who were recruited for the Salzburg Atherosclerosis Prevention Program (SAPHIR). The mitochondrial haplogroup [43] and CR polymorphism [29] data were obtained from previous studies.

### DNA isolation and mitochondrial DNA analysis

DNA was extracted from whole blood samples either by a modified salting out procedure as described by Miller et al. [44] or by using a NucleoSpin Blood Kit (Macherey-Nagel, Düren, Germany). A hierarchical system for mtDNA haplogrouping that combines multiplex PCR amplification, multiplex single-base primer extension, and capillary-based electrophoretic separation was used to assess the most common European haplogroups (H, U, J, T, K, I, V, W and X) as described in our pevious studies [45], [46]. Haplogroups that could not be assigned to one of the nine major European haplogroups by their single nucleotide polymorphism (SNP) combination were designated as “others”.

CR sequences were generated by direct DNA sequencing between nucleotide positions 16024 and 526. Polymerase chain reaction and sequencing was performed as described previously [45]; however, a different forward primer (15997f: CACCATTAGCACCCAAAGCT) was used. Data were analyzed with Chromas software 1.56 (Technelysium, Tewantin, Australia) and alignment was conducted with Blast 2 software (bl2seq) (http://blast.ncbi.nlm.nih.gov/Blast.cgi). The Cambridge Revised Sequence was used as a reference (GenBank accession number J01415).

### Statistical analysis

Frequencies of all mitochondrial haplogroups and CR polymorphisms were tested for independency from the disease using Pearson chi-square statistics and Fisher's exact test as appropriate. Only haplogroups and polymorphisms with a frequency ≥5% in either the melanoma or the control group were subjected to further statistical analysis. A p-value<0.05 was considered statistically significant. Association of A16183C, T16189C, C16192T, C16270T, and T195C with the disease state was adjusted for sex and age by logistic regression analysis.

The clinical parameters *mean Breslow thickness* and *metastasis* were analyzed within the melanoma cohort. Frequencies of all mitochondrial haplogroups and CR polymorphisms were tested for independency from the metastatic state using Pearson chi-square statistics and Fisher's exact test as appropriate. For comparison of mean Breslow thickness between different haplogroups and CR polymorphisms, Breslow data were logarithmically transformed and analyzed using a two-sample unpaired t-test. Adjustment for sex and age was conducted for A302CC-ins, T310C-ins, A73G and T16519C using logistic regression analysis.

All analyses were performed using PASW 18.0 (SPSS GmbH, Germany).

## Supporting Information

Table S1 **Control region polymorphisms of patients with melanoma.**
(DOCX)
