# Supplementary material for: Mitochondrial Haplogroups, Control Region Polymorphisms and Malignant Melanoma: A Study in Middle European Caucasians
Source: PLoS One. 2011 Dec 9;6(12):e27192. doi: 10.1371/journal.pone.0027192 (PMC3235102; doi:10.1371/journal.pone.0027192)
Supplement: Table S1 — Control region polymorphisms of patients with melanoma. (DOCX) [file pone.0027192.s001.docx]

## Supporting Information

**Table S1.** CR polymorphisms of patients with melanoma.

| Polymorphism in mtDNA control region | Frequency (%) in the melanoma cohort | n^1^ |
| --- | --- | --- |
| A16038G | 0.3 | 1 |
| A16051G | 1.1 | 4 |
| A16051C^2^ | 0.3 | 1 |
| C16069T | 11.4 | 40 |
| T16075C | 0.6 | 2 |
| T16092C | 1.4 | 5 |
| T16093C | 7.4 | 26 |
| C16104T | 0.3 | 1 |
| C16114A | 0.6 | 2 |
| C16114T | 0.3 | 1 |
| T16126C | 20.8 | 73 |
| G16129A | 5.7 | 20 |
| G16129C | 0.9 | 3 |
| C16134T | 0.6 | 2 |
| T16136C | 0.3 | 1 |
| T16140C | 0.6 | 2 |
| G16145A | 5.1 | 18 |
| C16147T | 0.6 | 2 |
| C16148T | 0.3 | 1 |
| G16153A | 1.1 | 4 |
| A16162G | 2.3 | 8 |
| A16163G | 3.1 | 11 |
| C16167T | 0.3 | 1 |
| C16168T | 1.1 | 4 |
| C16169T | 0.3 | 1 |
| T16172C | 2.0 | 7 |
| C16173T | 0.6 | 2 |
| C16176G | 0.9 | 3 |
| C16176T | 0.3 | 1 |
| C16179T | 2.0 | 7 |
| A16180del | 0.6 | 2 |
| A16182G | 0.3 | 1 |
| A16182C | 2.0 | 7 |
| A16183C | 5.7 | 20 |
| C16184A | 0.3 | 1 |
| C16186T | 3.7 | 13 |
| C16188T | 0.6 | 2 |
| T16189A | 0.3 | 1 |
| T16189C | 16.8 | 59 |
| C16192T | 9.7 | 34 |
| C16193T | 2.6 | 9 |
| C16201T | 0.3 | 1 |
| A16207G | 0.6 | 2 |
| T16209C | 1.4 | 5 |
| A16212G | 0.3 | 1 |
| G16213A | 0.3 | 1 |
| A16216G | 0.3 | 1 |
| A16219G | 0.3 | 1 |
| C16221T | 1.4 | 5 |
| C16222T | 1.4 | 5 |
| C16223T | 7.7 | 27 |
| T16224C | 4.8 | 17 |
| T16231C | 2.3 | 8 |
| A16233G | 0.9 | 3 |
| C16234T | 0.3 | 1 |
| A16235G | 0.3 | 1 |
| C16239T | 0.9 | 3 |
| C16248T | 1.7 | 6 |
| T16249C | 0.9 | 3 |
| G16255A | 0.6 | 2 |
| C16256T | 7.7 | 27 |
| C16257T | 0.3 | 1 |
| A16258T | 0.3 | 1 |
| A16258C | 0.6 | 2 |
| C16259T | 0.3 | 1 |
| C16260T | 0.3 | 1 |
| C16261T | 4.0 | 14 |
| C16262T | 0.3 | 1 |
| T16263C | 0.3 | 1 |
| C16264T | 0.3 | 1 |
| A16265G | 0.9 | 3 |
| C16266T | 0.9 | 3 |
| A16269G | 0.6 | 2 |
| C16270T | 12.5 | 44 |
| T16271C | 0.9 | 3 |
| G16274A | 0.9 | 3 |
| C16278T | 3.1 | 11 |
| C16287A | 0.3 | 1 |
| T16288C | 0.3 | 1 |
| C16290T | 0.9 | 3 |
| C16291T | 3.7 | 13 |
| C16292T | 2.8 | 10 |
| A16293G | 2.3 | 8 |
| C16294T | 10.0 | 35 |
| C16295T | 0.9 | 3 |
| C16296T | 4.6 | 16 |
| T16297C | 0.6 | 2 |
| T16298C | 4.0 | 14 |
| A16300G | 0.9 | 3 |
| C16301T | 0.6 | 2 |
| T16304C | 6.8 | 24 |
| A16309G | 0.9 | 3 |
| T16311C | 10.3 | 36 |
| A16316G | 1.4 | 5 |
| G16319A | 1.7 | 6 |
| C16320T | 1.4 | 5 |
| T16324C | 0.3 | 1 |
| T16325C | 2.3 | 8 |
| C16327A | 0.6 | 2 |
| C16327T | 0.3 | 1 |
| A16343G | 1.7 | 6 |
| T16352C | 0.6 | 2 |
| C16354T | 0.9 | 3 |
| C16355T | 1.1 | 4 |
| T16356C | 4.6 | 16 |
| T16359C | 0.3 | 1 |
| T16362C | 5.1 | 18 |
| T16368C | 0.6 | 2 |
| C16380T | 0.3 | 1 |
| G16390A | 1.7 | 6 |
| G16391A | 1.7 | 6 |
| G16398A | 1.1 | 4 |
| A16399G | 6.0 | 21 |
| C16400T | 0.3 | 1 |
| G16428A | 0.3 | 1 |
| A16463G | 0.3 | 1 |
| G16477T^2^ | 0.3 | 1 |
| A16482G | 0.9 | 3 |
| G16485A^2^ | 0.9 | 3 |
| T16489C^2^ | 0.9 | 3 |
| T16491C | 0.9 | 3 |
| G16496A^2^ | 0.9 | 3 |
| A16497G | 0.3 | 1 |
| T16519C | 63.5 | 223 |
| A16524G | 0.3 | 1 |
| G16526A | 2.0 | 7 |
| C16527T | 1.4 | 5 |
| C61T | 0.3 | 1 |
| G62A | 0.3 | 1 |
| T63C | 0.3 | 1 |
| T72C | 2.0 | 7 |
| A73G | 54.7 | 192 |
| A93G | 2.3 | 8 |
| T119C | 0.3 | 1 |
| T131C | 0.3 | 1 |
| G143A | 0.6 | 2 |
| T146C | 10.3 | 36 |
| C150T | 11.1 | 39 |
| C151T | 0.3 | 1 |
| T152C | 23.6 | 83 |
| A153G | 1.4 | 5 |
| T158C^2^ | 0.3 | 1 |
| A183G | 0.6 | 2 |
| G184A | 0.3 | 1 |
| G185A | 6.3 | 22 |
| A188G | 0.9 | 3 |
| A189G | 3.4 | 12 |
| C194T | 1.7 | 6 |
| T195C | 21.9 | 77 |
| C198T | 0.9 | 3 |
| T199C | 2.8 | 10 |
| A200G | 1.4 | 5 |
| T204C | 5.1 | 18 |
| G207A | 4.3 | 15 |
| A214G | 0.3 | 1 |
| A215G | 2.6 | 9 |
| T217C | 0.9 | 3 |
| C222T | 0.6 | 2 |
| G225A | 1.4 | 5 |
| G225T^2^ | 0.6 | 2 |
| T226C | 0.6 | 2 |
| A227G | 0.6 | 2 |
| A227T | 0.6 | 2 |
| G228A | 6.8 | 24 |
| G228T | 0.9 | 3 |
| A234T^2^ | 0.6 | 2 |
| T239C | 2.6 | 9 |
| A240G | 0.3 | 1 |
| A240T^2^ | 0.6 | 2 |
| C242T | 1.1 | 4 |
| G247A | 0.6 | 2 |
| G247T^2^ | 0.6 | 2 |
| A248del | 0.3 | 1 |
| A249T^2^ | 0.6 | 2 |
| T250C | 1.7 | 6 |
| A257G | 0.9 | 3 |
| A259G | 0.3 | 1 |
| A263G | 0.3 | 1 |
| T279C | 0.6 | 2 |
| C295T | 10.5 | 37 |
| C296T | 0.3 | 1 |
| A302C-ins | 39.6 | 139 |
| A302CC-ins | 12.0 | 42 |
| T310C | 0.0 | 0 |
| T310TC-ins | 1.7 | 6 |
| T310C-ins | 98.3 | 345 |
| G316A | 0.3 | 1 |
| T319C | 2.6 | 9 |
| C340T | 0.6 | 2 |
| C345T | 0.3 | 1 |
| A396G^2^ | 0.3 | 1 |
| T398del^2^ | 0.3 | 1 |
| T408A | 0.3 | 1 |
| T452del | 0.6 | 2 |
| C456T | 2.0 | 7 |
| C462T | 8.0 | 28 |
| T477C | 1.4 | 5 |
| T482C | 0.9 | 3 |
| T489C | 12.3 | 43 |
| C494del | 0.3 | 1 |
| C497T | 1.4 | 5 |
| G499A | 3.7 | 13 |
| C506T | 0.6 | 2 |
| A508G | 0.9 | 3 |
| G513A | 2.8 | 10 |
| G513CA-ins | 3.7 | 13 |
| G513CACA-ins | 2.0 | 7 |
| G514CACACA-ins | 0.9 | 3 |
| C514del | 6.8 | 24 |
| C514GC-ins^2^ | 0.3 | 1 |
| A515del | 7.1 | 25 |

^1^ n = Number of individuals with the respective polymorphism.

^2^ Polymorphisms not listed in MITOMAP or the Human Mitochondrial Genome Database.
